# Psychopathological and neuropsychological disorders associated with chronic primary visceral pain: Systematic review

**DOI:** 10.3389/fpsyg.2022.1031923

**Published:** 2022-10-19

**Authors:** Alejandro Arévalo-Martínez, Juan Manuel Moreno-Manso, María Elena García-Baamonde, Macarena Blázquez-Alonso, Pilar Cantillo-Cordero

**Affiliations:** Department of Psychology, University of Extremadura, Badajoz, Spain

**Keywords:** chronic primary pain, chronic primary visceral pain, psychopathology, neuropsychology, systematic review

## Abstract

The World Health Organization (WHO), in its last review of its International Classification of Diseases, established a new classification for chronic pain. Among the principal categories, of particular interest is chronic primary pain as a new type of diagnosis in those cases in which the etiology of the disease is not clear, being termed as chronic primary visceral pain when it is situated in the thorax, abdomen, or pelvis. Due to the novelty of the term, the objective of the systematic review was to examine the psychopathological and neuropsychological disorders associated with chronic primary visceral pain. We carried out a search of the scientific literature following the PRISMA directives using the Pubmed, Medline, PsycInfo and Scopus databases. A total of 33 articles were selected after applying the inclusion and exclusion criteria. The analysis of the studies showed that most persons with chronic primary visceral pain suffer from at least one psychological disorder; the most prevalent being anxiety, depressive or somatoform disorders. The most frequent psychopathological symptoms are anxiety, depression and somatization. Similarly, the findings are insufficient to determine the existence of deficits in the domains of executive functioning, memory and intelligence. However, the existence of attention biases does seem to be clear. This review supposes a starting point for conceptualizing chronic primary visceral pain. It is necessary to continue further research so as to obtain a better understanding of this pathology and the disorders associated.

## Introduction

Chronic pain is one of the most frequent health problems in the adult population, with a variable prevalence of between 11 and 50% worldwide (De Souza et al., 2017; Andrews et al., 2018; Murray et al., 2021). It is a prolonged and severe multifactorial illness involving biological, psychological, and social factors (Vargas et al., 2018; Korwisi et al., 2021). The World Health Organization (WHO) together with the International Association for the Study of Pain (IASP), in their last review of the International Classification of Diseases (ICD-11), defined chronic pain as “an unpleasant sensory and emotional experience associated with, or resembling that associated with, actual or potential tissue damage. Chronic pain is pain that persists or recurs for longer than 3 months” (World Health Organization, 2018, MG30 Chronic Pain).

The research carried out over the last few decades has provided evidence of the multiple consequences of chronic pain on a psychopathological and neuropsychological level (Fine, 2011; Mills et al., 2019; Cáceres-Matos et al., 2020). From the psychopathological point of view, the presence of chronic pain is associated with a high probability of developing psychological disorders, depression, anxiety and sleep disorders being those that present the greatest co-morbidity (Arango-Dávila and Rincón-Hoyos, 2018; Cáceres-Matos et al., 2020); while from the neuropsychological point of view, the most common cognitive difficulties occur in the domains of attention, memory, intelligence and the executive functioning (Berryman et al., 2014; Corti et al., 2021). Similarly, such variables as age, the presence of anxiety or depression, sleep disorders, the use of medication and the type, intensity, and duration of the pain, among others, would seem to have a significant influence on the cognitive performance of persons suffering from chronic pain (Moriarty et al., 2017; Ojeda et al., 2018).

**Figure 1:**
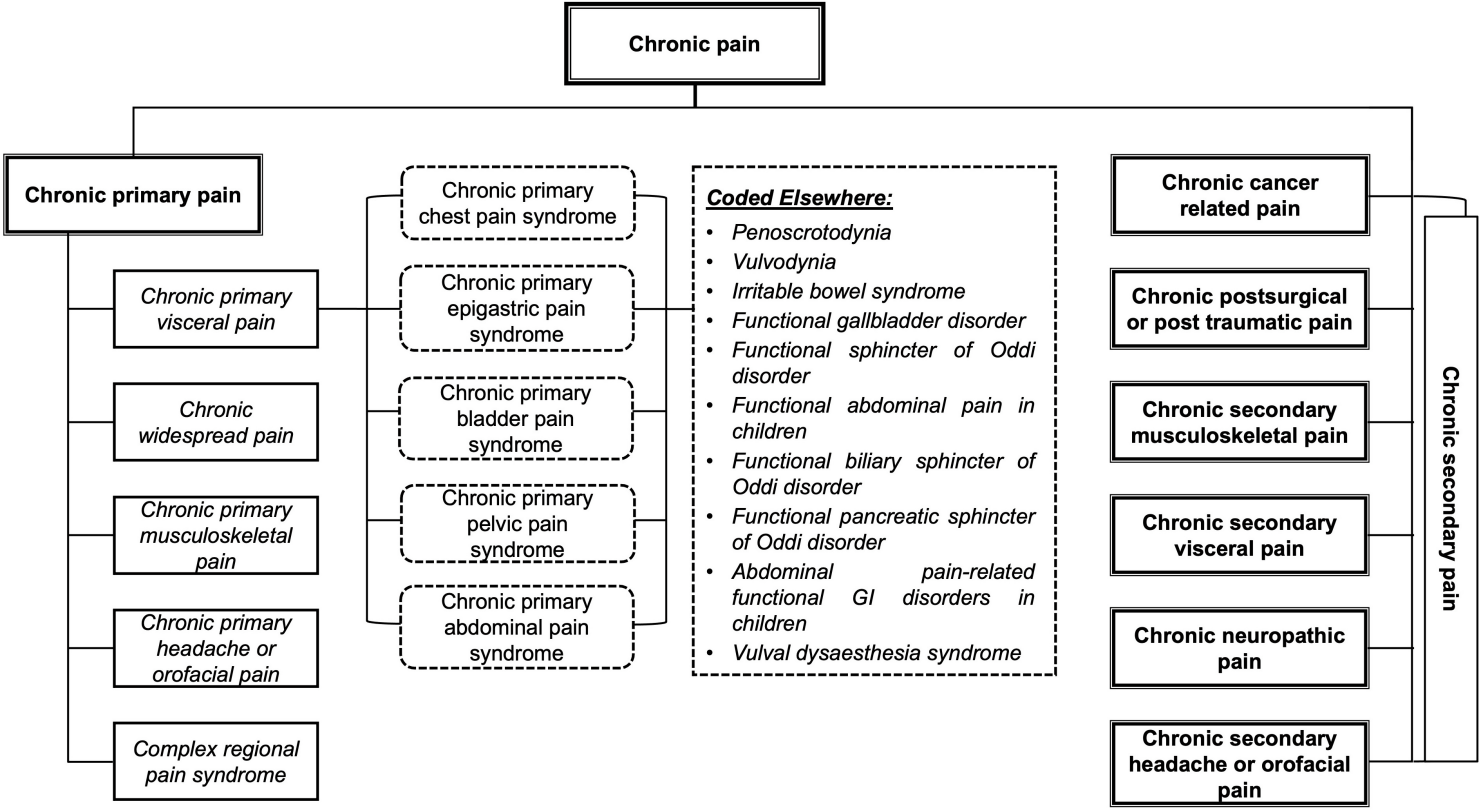
Classification of chronic pain: chronic primary pain and chronic secondary pain. Version adapted from Treede et al. (2019).

The integral classification of the ICD-11 allows us to approach chronic pain from a biopsychosocial point of view, establishing seven main diagnostic categories that, in turn, arte subdivided into specific diagnostic subcategories (World Health Organization, 2018; Korwisi et al., 2021). In the first category, called chronic primary pain, no underlying disease or known harmful process can explain the symptoms of the chronic pain; while, in the six remaining categories, called chronic secondary pain, the underlying disease or known harmful process does explain the symptoms of the chronic pain (see Figure 1) (Treede et al., 2019; Korwisi et al., 2021).

Chronic primary pain is a new kind of diagnosis that aims to redress the deficiencies identified in the previous versions of the ICD, allowing a diagnosis when the etiology is not clear, but there is evidence of significant emotional distress and/or functional impairment (Nicholas et al., 2019). It can be defined as chronic pain present in one or various anatomical regions associated with significant emotional distress and/or functional impairment that affect the basic, instrumental, and advanced activities of daily life (World Health Organization, 2018; Treede et al., 2019). It is the result of the interaction between biological, psychological, and social factors, yet it can be diagnosed independently of the influence of the first two, except when a different diagnosis can better explain the symptoms (Nicholas et al., 2019). This approach goes beyond considering chronic primary pain as a symptom and starts to consider it as a health condition or prolonged illness in itself (Nicholas et al., 2019; Treede et al., 2019).

Within the five subcategories of chronic primary pain, chronic primary visceral pain (CPVP) represents an important condition that, as do the other subcategories, negatively affects the life of a person and the different contexts in which that person's life is led, be it social life, family life or work life, and is associated with a low quality of life, as well as problems with both physical and psychological health (Dueñas et al., 2016; Vargas et al., 2018; Cáceres-Matos et al., 2020). In order to define CPVP^1^, the ICD-11 uses the following criteria (World Health Organization, 2018):

(A) Chronic primary pain.
(B) Situated in the thoracic, abdominal or pelvic regions.
(C) Associated with significant emotional distress and/or functional impairment.
(D) The specific anatomical situation of the pain is compatible with the typical pain coming from specific internal organs.

Although the existing relations between the diagnosis of chronic pain and the development of psychopathological and neuropsychological disorders seem to display sufficient evidence, the conceptualization of chronic primary pain as a new diagnostic category presents numerous practical implications that should be explored. One such implication is the possibility of identifying the disorders on a biological, psychological and social level associated with each one of the diagnostic subcategories and, from there, developing specific multidisciplinary treatments for the existing deficits (Nicholas et al., 2019). In this sense, despite the fact that many works of research have evaluated the psychological disorders associated with chronic pain in an isolated manner (Fine, 2011; Riegel et al., 2014; De Souza et al., 2017; Moriarty et al., 2017; Arango-Dávila and Rincón-Hoyos, 2018; Ojeda et al., 2018; Racine, 2018; Vargas et al., 2018; Corti et al., 2021), neither the systematic reviews nor the meta-analyses currently published (Berryman et al., 2014; Riegel et al., 2014; Dueñas et al., 2016; Mills et al., 2019; Cáceres-Matos et al., 2020) have been able to identify the psychopathological and neuropsychological disorders in each one of the subcategories of chronic primary pain, in accordance with the new classification proposal by the ICD-11.

Thus, the objective of this present review is to critically describe and analyse the works published in the last 10 years concerning the psychopathological and neuropsychological evaluation of CPVP, using the inclusion criteria established by the WHO in the ICD-11 to do so.

## Method

### Search and inclusion criteria

The bibliographic material was selected from journals, but not PhDs or chapters from books, indexed over the last 10 years (January 2012 – May 2022), in English or Spanish, through the following databases: Pubmed, Medline, PsycInfo and Scopus. Given the existence of different diagnostic subtypes in the CPVP and their relation to the disorders under review, we carried out a combined search using the following terms: (Chronic primary visceral pain OR Chronic visceral pain OR Chronic primary chest pain syndrome OR Chronic primary epigastric pain syndrome OR Chronic primary bladder pain syndrome OR Chronic primary pelvic pain syndrome OR Chronic primary abdominal pain syndrome OR Penoscrotodynia OR Vulvodynia OR Irritable bowel syndrome OR Functional gallbladder disorder OR Functional sphincter of Oddi disorder OR Functional biliary sphincter of Oddi disorder OR Functional pancreatic sphincter of Oddi disorder OR Vulval dysaesthesia syndrome) AND (psychopathology OR mental disorder OR mental illness OR neuropsychology OR neuropsychological test OR neuropsychological assessment OR cognitive impairment OR cognitive dysfunction OR cognitively impaired OR executive function OR cognitive function OR cognitive performance OR memory OR attention).

In order to be included in this review, the publications had to comply with the following additional criteria: (a) they must be empirical works of research evaluating psychopathological and/or neuropsychological disorders in any of the subtypes of CPVP; (b) they must have a sample of adult persons between 18 and 85 years of age; (c) the CPVP must not be the consequence of an illness; (d) the sample must not have additional illnesses; (e) they must use psychological and/or neuropsychological evaluation tests; (f) they must include the results of the psychological and/or neuropsychological evaluation in the base line; (g) they must include the necessary data for determining the existence of psychopathological and/or neuropsychological disorders in the sample.

As exclusion criteria, we discarded: (a) publications in which the abstract made no reference to CPVP or any of the subtypes or the related psychopathological and/or neuropsychological variables; (b) publications that did not specify the subtype of CPVP or in which, when specified, these did not form part of the objectives of the publication.

### Codification of the studies

We set out a series of variables concerning the reviewed publications related to the design and methodology of the study: (a) the country in which the research was carried out, (b) the design of the study, (c) the subtypes of CPVP, (d) the existence of a control group and/or other additional groups, (e) the number of participants, (f) the gender and age of the participants, (g) the instruments used to evaluate the CPVP and the psychopathological and neuropsychological disorders, (h) the variables of the CPVP, both psychopathological and neuropsychological, (i) and the results of the evaluation of the CPVP and the psychopathological and neuropsychological disorders.

## Results

### Selection of the studies and the characteristics

**Figure 2:**
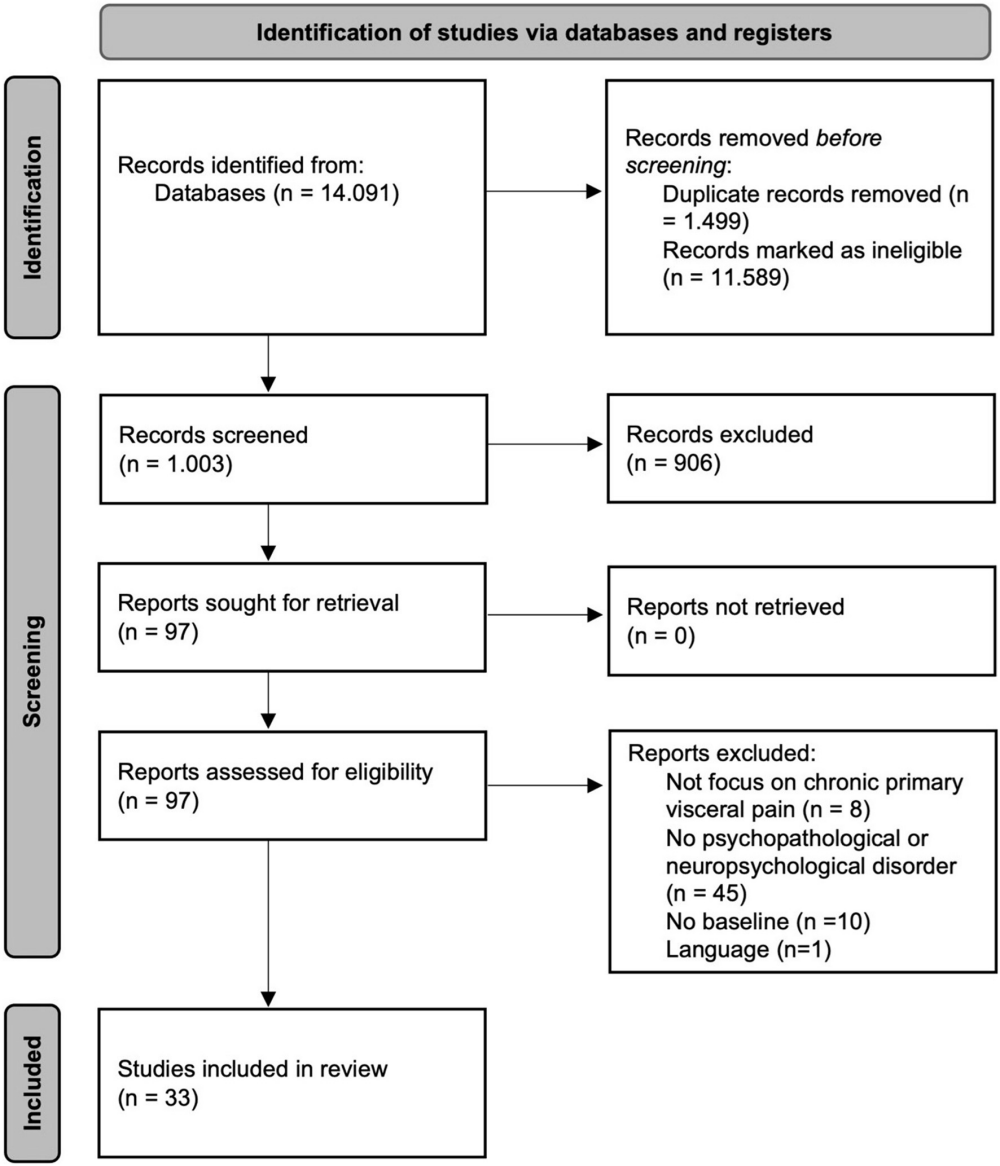
PRISMA flow diagram (Page et al., 2021).

From the results of the bibliographic search, we identified a total of 14,091 articles. After eliminating 1,499 duplicated articles and 11,589 more that were unrelated to the subject matter, we reviewed 1,003 articles based on the title and content of the abstract. Of these, we excluded a further 906 articles as they did not adjust to the objective of the review. A total of 97 articles concerning psychopathological and neuropsychological disorders were taken into consideration for the review, of which 64 were excluded following a reading of the complete text as they did not comply with the inclusion criteria. A total of 33 articles were finally included in the present review. The details are shown in the PRISMA flow diagram of Figure 2.

The countries in which the research work of the 33 selected articles was carried out show a high degree of heterogeneity, but the major origins were Germany (15%), the United States (12%), China, India and Italia (9% each). These were followed by Iran, the United Kingdom and Turkey (6% each), as well as Australia, Canada, South Korea, Croatia, France, Ireland, Japan and Norway (3% each).

### Design of the study, CPVP subtypes and the existence of a control group and/or additional groups

Table 1 presents the principal characteristics of the articles included in the review. The rest of the variables, referring to psychopathological and neuropsychological disorders respectively, can be seen in Tables 2, 3. The methodological design followed by the majority of the research works is the cross-sectional study (82%), while the rest use a longitudinal study (18%).

**Table 1:** Characteristics of the studies included in the review.

**References**	**Country**	**Design**	**Subtypes CPVP**
Aizawa et al. (2012)	Japan	Cross-sectional study	Irritable bowel syndrome (IBS)
Sertbas et al. (2012)	Turkey	Cross-sectional study	Irritable bowel syndrome (IBS)
Berrill et al. (2013)	The UK	Cross-sectional study	Irritable bowel syndrome (IBS)
Lackner et al. (2013)	The USA	Cross-sectional study	Irritable bowel syndrome (IBS)
van Tilburg et al. (2013)	The USA	Cross-sectional study	Irritable bowel syndrome (IBS)
Kennedy et al. (2014)	Ireland	Cross-sectional study	Irritable bowel syndrome (IBS)
Phillips et al. (2014)	Australia	Cross-sectional study	Irritable bowel syndrome (IBS)
Tkalcic et al. (2014)	Croatia	Cross-sectional study	Irritable bowel syndrome (IBS)
Farup and Hestad (2015)	Norway	Cross-sectional study	Irritable bowel syndrome (IBS)
Hubbard et al. (2015)	The USA	Cross-sectional study	Irritable bowel syndrome (IBS)
Lee et al. (2015)	China	Longitudinal study	Irritable bowel syndrome (IBS)
Liu et al. (2015)	China	Longitudinal study	Irritable bowel syndrome (IBS)
Chen et al. (2016)	China	Longitudinal study	Irritable bowel syndrome (IBS)
Thakur et al. (2016)	The USA	Cross-sectional study	Irritable bowel syndrome (IBS)
Banerjee et al. (2017)	India	Cross-sectional study	Irritable bowel syndrome (IBS)
Baniasadi et al. (2017)	Iran	Cross-sectional study	Irritable bowel syndrome (IBS)
Brünahl et al. (2017)	Germany	Cross-sectional study	Chronic primary pelvic pain syndrome (CPPPS)
Kawoos et al. (2017)	India	Cross-sectional study	Irritable bowel syndrome (IBS)
Lee et al. (2017)	South Korea	Cross-sectional study	Irritable bowel syndrome (IBS)
Bruno et al. (2018)	Italy	Cross-sectional study	Irritable bowel syndrome (IBS)
Dybowski et al. (2018)	Germany	Longitudinal study	Chronic primary pelvic pain syndrome (CPPPS)
Henrich and Martin (2018)	The UK	Cross-sectional study	Irritable bowel syndrome (IBS)
Berens et al. (2019)	Germany	Cross-sectional study	Irritable bowel syndrome (IBS)
Piontek et al. (2019)	Germany	Cross-sectional study	Chronic primary pelvic pain syndrome (CPPPS)
Stasi et al. (2019)	Italia	Longitudinal study	Irritable bowel syndrome (IBS)
Wong et al. (2019a)	China	Cross-sectional study	Irritable bowel syndrome (IBS)
Bouchoucha et al. (2020)	France	Longitudinal study	Irritable bowel syndrome (IBS)
Klotz et al. (2020)	Germany	Cross-sectional study	Chronic primary pelvic pain syndrome (CPPPS)
Porcelli et al. (2020)	Italy	Cross-sectional study	Irritable bowel syndrome (IBS)
Rustamov et al. (2020)	Canada	Cross-sectional study	Irritable bowel syndrome (IBS)
Torun et al. (2020)	Turkey	Cross-sectional study	Irritable bowel syndrome (IBS)
Gogheri et al. (2021)	Iran	Cross-sectional study	Irritable bowel syndrome (IBS)
Sharma et al. (2021)	India	Cross-sectional study	Irritable bowel syndrome (IBS)

**Table 2:** Psychopathological disorders.

**References**	**Sample**	**Gender and age**	**Instruments**	**Results**
Sertbas et al. (2012)	*n* = 100 - IBS (50) - Control (50)	Male = 32 (32%) Female = 68 (68%) Mean age=37.35	Structured Clinical Interview for DSM-IV (SCID-I/SCID-II) Beck Depression Inventory (BDI) Beck Anxiety Inventory (BAI) Symptom Check list – 90 (Revised) [SCL-90-R]	The group with IBS presents a total severity index (*p* < 0.01), somatization (*p* < 0.01), compulsive obsession (*p* < 0.01), interpersonal sensitivity (*p* < 0.01), depression (*p* < 0.01), anxiety (*p* < 0.01), hostility (*p* < 0.01), phobias (*p* = 0.01), paranoid ideation (*p* < 0.01) and psychoticism (*p* = 0.01) significantly higher than the control group. Thirty-four percent of the IBS patients have at least one diagnosis from the Axis-I (*p* < 0.01) and 14% from the Axis-II (not significant), the most prevalent being anxiety disorders (12%), mood disorders (8%), somatoform disorders (6%), narcissistic personality disorder (4%), obsessive-compulsive disorder (4%), borderline personality disorder (2%), dependent personality disorder (2%) and histrionic personality disorder (2%).
Lackner et al. (2013)	*n* = 175	Male = 38 (21.7%) Female = 137 (78.3%) Mean age = 41	National Health Interview Survey (NHIS) MINI-International Neuropsychiatric Interview (MINI) - Computerized version IBS Symptom Severity Scale (IBS-SSS) Brief Symptom Inventory (BSI-18) IBS-Quality of Life (IBS-QOL) 12-Item Short Form Health Survey (SF-12)	The sample with IBS presents high levels of psychological distress and severity of gastrointestinal symptoms, a significant deterioration in the quality of life, as well as deterioration in physical and psychological functioning. Forty-seven percent have a psychological comorbidity disorder and 26% have at least two, the most common being anxiety disorders (69%) and affective disorders (38%); while 31% have a physical comorbidity disease and 71% have at least two. The physical and psychological comorbidities were moderately correlated (r = 0.17; *p* < 0.05) and, furthermore, they also correlated with a worse quality of life related to the illness (r = 0.28; r = −0.30) and overall distress (r = −0.21; r = −0.64).
van Tilburg et al. (2013)	*n* = 286	Male = 53 (18.5%) Female = 233 (81.5%) Mean age = 34.6	IBS Symptom Severity Scale (IBS-SSS) Sexual and Physical Abuse Questionnaire (SPAHQ) Family Inventory of Life Events scale (FILE) NEO Personality Inventory (NEO PI-R) Brief Symptom Inventory (BSI-18) Catastrophizing scale of the Coping Strategies Scale	The sample with IBS presents moderate (43%) to severe (39.5%) gastrointestinal symptoms. The model that best predicts the variation in the severity of the symptoms of the IBS, to be precise 36%, includes only the variables of catastrophizing (β = 0.33; *p* < 0.001) and somatization (β = 0.20; *p* < 0.001). Nevertheless, such variables as anxiety (β = 0.80; *p* < 0.001) and somatization (β = 0.37; *p* < 0.001) have an indirect effect on the symptoms of the IBS through catastrophizing. Furthermore, the presence of neuroticism (β = 0.66; *p* < 0.001) and stressful life events (β = 0.31; *p* < 0.001) predict the presence of anxiety.
Lee et al. (2015)	*n* = 23.445 - IBS (4.689) - Control (18.576)	Male = 13.235 (56.45%) Female = 10.210 (43.55%) Mean age = 47.47	Clinical diagnoses from the Longitudinal Health Insurance Database 2005 (LHID 2005) - General Psychopathology Assessment	The group with IBS presents a significantly higher prevalence than the control group in the following disorders: depressive, anxiety, sleep and bipolar. In the period the group was being followed, there was a higher general incidence rate than the control group in the depressive disorder (4.70/1.74 per 1,000 persons/year), anxiety disorder (4.00/1.39 per 1,000 persons/year), sleep disorder (2.94/1 per 1,000 persons/year), bipolar disorder (0.32/0.13 per 1,000 persons/year) and schizophrenia (0.25/0.14 per 1,000 persons/year).
Liu et al. (2015)	*n* = 61.592 - IBS (30.796) - Control (30.796)	Male = 29.072 (47.2%) Female = 32.520 (52.8%) Mean age= 50	Clinical diagnoses from the Taiwan's National Health Insurance Research Database (NHIRD) - Bipolar Disorder Assessment	In the IBS group, the general incidence rate for bipolar disorder was significantly higher than for the control group (1.6/0.6 per 1,000 persons/year). The group with IBS also presented a significantly greater risk of developing bipolar disorder than the control group and, in addition, the risk of developing bipolar disorder is greater in those IBS patients with a greater number of years being followed.
Thakur et al. (2016)	*n* = 384	Male = 77 (20.1%) Female = 307 (79.9%) Mean age = 41.50	IBS Medical Comorbidity - Comorbidity checklist that covers 112 medical conditions Brief Symptom Inventory (BSI-18) IBS Symptom Severity Scale (IBS-SSS) IBS-Quality of Life (IBS-QOL)	The sample with IBS presents a severity of gastrointestinal symptoms from moderate to severe, high levels of anxiety and depression, and a low quality of life related to the illness. - The number of medical comorbidities correlated positively with the duration of the IBS symptoms (r = 0.35), anxiety (r = 0.17), depression (r = 0.21) and the severity of the symptoms (r = 0.11); it also correlated negatively with the quality of life (r = −0.15). - The high levels of anxiety were associated with higher levels of depression (r = 0.68), a greater severity of the symptoms (r = 0.17) and a lower quality of life (r = −0.37). - The high levels of depression were associated with a greater severity of the symptoms (r = 0.22) and a lower quality of life (r = −0.43).
Banerjee et al. (2017)	*n* = 100 - IBS (50) - Control (50)	Male = 74 (74%) Female = 26 (26%) Mean age = 37.49	Hamilton Rating Scale for Depression (HAM-D) Hamilton Rating Scale for Anxiety (HAM-A) IBS Symptom Severity Scale (IBS-SSS)	The group with IBS presents moderate levels of depression (*p* < 0.001) and anxiety (*p* < 0.001), there being significant differences with respect to the control group. Among those patients with IBS, 26% present a moderate severity of gastrointestinal symptoms and 74% severe symptoms. The patients with moderate IBS present low levels of depression and anxiety, and those with severe IBS present moderate levels of depression and severe levels of anxiety; there are significant differences between both in anxiety (*p* < 0.001), but not in depression (*p* = 0.062).
Baniasadi et al. (2017)	*n* = 123	Male = 48 (39%) Female = 75 (61%) Mean age = 29	Rome III Criteria for IBS Depression Anxiety Stress Scales (DASS) Pittsburgh Sleep Quality Index (PSQI)	The sample with IBS was classified into the subtypes IBS-Constipation (38%), IBS-Diarrhea (42.3%) and IBS-Mixed (19.5%). 70.2% of the sample present depression, 75.63% anxiety and 78.86% stress, while the quality of sleep was slightly deteriorated. In addition, depression (*p* = 0.034), anxiety (*p* = 0.011) and stress (*p* = 0.029) are significantly higher in those subjects with a worse quality of sleep.
Brünahl et al. (2017)	*n* = 178	Male = 71 (39.9%) Female = 107 (60.1%) Mean age = 49.1	Structured Clinical Interview for DSM-IV (SCID-I) Modules of the Patient Health Questionnaire (PHQ-D): Patient Health Questionnaire Depression Scale (PHQ-9) Generalized Anxiety Disorder Scale (GAD-7) Patient Health Questionnaire (PHQ-15) for the evaluation of somatic symptom severity Visual Analog Scale (VAS) Short-form McGill Pain Questionnaire (SF-MPQ) NIH-Chronic Prostatitis Symptom Index (NIH-CPSI)	In the sample with CPPPS, 95.2% comply with the criteria to be able to diagnose them with at least one psychological disorder. To be precise, 31.5% comply with the criteria for being diagnosed as having one psychological disorder, 30.4% as having two, 16.7% three and 16.7% four. - 91.7% comply with the criteria for having a somatoform disorder. Pain disorder (p < 0.001), somatization disorder (*p* < 0.001) and hypochondria (*p* < 0.001) are significantly higher than in the general population. - 50.6% comply with the criteria for having mood disorder. The major depressive disorder (*p* < 0.001) and the persistent depressive disorder (*p* < 0.001) are significantly higher than in the general population. - 32.1% comply with the criteria for having anxiety disorder. The panic disorder (*p* < 0.001) and the generalized anxiety disorder (*p* < 0.001) are significantly higher than in the general population. - 8.9% comply with the criteria for having substance use disorder. The severity of somatic symptoms (*p* < 0.001), depression (*p* < 0.001) and anxiety (*p* < 0.001) in CPPPS are significantly higher than in the general population.
Kawoos et al. (2017)	*n* = 360 - IBS (160) - Control (200)	Male = 118 (32.8%) Female = 242 (67.2%) Mean age= 38.7	Rome III Criteria for IBS - MINI-International Neuropsychiatric Interview (MINI-Plus)	The group with IBS was classified into the subtypes IBS-Constipation (25%), IBS-Diarrhea (22.5%), IBS-Mixed (42.5%) and IBS-Not specified (10%). In the IBS group, 84.4% presented some kind of psychological disorder, as opposed to 41.5% of the control group. In the IBS group, the presence of the generalized anxiety disorder (*p* = 0.011), major depression (*p* < 0.001), mixed anxiety-depression (*p* < 0.001) and others, such as the panic disorder, adaptation disorder and dysthymia (*p* = 0.037) were significantly higher than in the control group. The most prevalent disorders in the IBS group were the generalized anxiety disorder (30%) and the major depressive disorder (27.5%).
Lee et al. (2017)	*n* = 3.429 - IBS (374) - Control (3.055)	Male = 1,789 (52.2%) Female = 1,640 (47.8%) Mean age = 52.6	Rome III Criteria for IBS - Beck Depression Inventory (BDI) - Self-report measures of insomnia - Questionnaire on lifestyle and anthropometric measurements	The total sample was divided into terciles with respect to the BDI score. The proportion of patients with IBS becomes greater the worse the depressive symptoms are, the probabilities of suffering from IBS being 1.61 (*p* < 0.0021) in the middle tercile and 2.55 (*p* < 0.0001) in the upper tercile as compared to the control group; furthermore, those persons suffering from insomnia had an 81% greater probability of having IBS (*p* < 0.001); while the probabilities of IBS were significantly higher in all the terciles with insomnia (*p* < 0.001).
Bruno et al. (2018)	*n* = 111 - IBS– Constipation Subtype (34) - IBS– Diarrhea Subtype (37) - IBS– Mixed Subtype (40)	Male = 48 (43.2%) Female = 63 (56.8%) Mean age = 46.6	Hamilton Rating Scale for Depression (HAM-D) - Hamilton Rating Scale for Anxiety (HAM-A) - State-Trait Anger Expression Inventory-2 (STAXI-2)	The sample with IBS presents moderate levels of depression in IBS-Constipation and slight levels in IBS-Diarrhea and IBS-Mixed, without there being significant differences between them. It shows slight to moderate levels of anxiety in IBS-Constipation and slight in IBS-Diarrhea and IBS-Mixed, and there are significant differences between them. Finally, it also shows levels of expression of rage state-trait within the normal range in IBS-Constipation, IBS-Diarrhea and IBS-Mixed; there are also significant differences between them in the subscales of anger (*p* < 0.001) and angry (*p* < 0.002).
Dybowski et al. (2018)	*n* = 109	Male = 44 (40.4%) Female = 65 (59.6%) Mean age = 49.3	NIH-Chronic Prostatitis Symptom Index (NIH-CPSI) - Patient Health Questionnaire Anxiety and Depression Scale (PHQ-ADS): - Pain Catastrophizing Scale (PCS) - Whiteley Index-7 (WI-7) - Perceived Social Support Questionnaire (F-SozU)	The sample with CPPPS presents moderate levels in the severity of the symptoms. To be precise, it shows moderate levels in the subscales of pain, slight in urinary symptoms and moderate to severe in quality of life. The levels of depression and anxiety are slight, those of pain catastrophizing are slight to moderate and those of social support are high. The severity of the pain (β = 0.29; *p* = 0.004), the anxiety-depressive symptomatology (β = 0.29; *p* = 0.009), the urinary symptoms (β = 0.24; *p* = 0.01) and age (β = 0.27; *p* = 0.02) are all significant predictors of the evolution of the pain; while the anxiety-depressive symptomatology is a significant predictor (β = 0.27; *p* = 0.01) of the deterioration in the quality of life.
Berens et al. (2019)	*n* = 381 - IBS (127) - IBD (127) - Control (127)	Male = 140 (37%) Female = 240 (63%) Mean age = 35.6	IBS Symptom Severity Scale (IBS-SSS) - Manitoba Inflammatory Bowel Disease Index (MIBDI) - Visual Analog Scale (VAS) of The EuroQol-5D Somatic Symptom Scale - 8 (SSS-8) - Patient Health Questionnaire Depression Scale (PHQ-9) - Generalized Anxiety Disorder Scale (GAD-7) - Whiteley Index-7 (WI-7) - Adverse Childhood Experiences (ACEs) Experiences in Close Relationships Scale (ECR-RD12) - Mentalizing Questionnaire (MZQ)	The group with IBS presents a significantly worse state of health (*p* < 0.001) and severity of gastrointestinal symptoms (*p* < 0.001) than the control group. In addition, it presents significantly high levels of somatization (*p* < 0.001/ *p* < 0.001), depressive symptoms (*p* < 0.001/ *p* = 0.002), anxiety symptoms (*p* < 0.001/ *p* < 0.001) and anxiety due to illness (*p* < 0.001/*p* < 0.001) in comparison to the control group and to the group of inflammatory bowel diseases (IBD). As for the psychological risk factors, these show a significantly higher proportion of adverse childhood experiences (*p* = 0.048), insecure styles of attachment (*p* = 0.039) and mentalizing deficits (*p* < 0.001) in comparison to the control group, as well as a significantly higher proportion of adverse childhood experiences (*p* = 0.048) and mentalizing deficits (*p* < 0.017) in comparison to the inflammatory bowel diseases group.
Piontek et al. (2019)	*n* = 234	Male = 103 (44%) Female = 131 (56%) Mean age = 47.93	NIH-Chronic Prostatitis Symptom Index (NIH-CPSI) - Short-form McGill Pain Questionnaire (SF-MPQ) - 12-Item Short Form Health Survey (SF-12) Patient Health Questionnaire Depression Scale (PHQ-9) - Generalized Anxiety Disorder Scale (GAD-7) - Pain Catastrophizing Scale (PCS) - Perceived Social Support Questionnaire (F-SozU)	The sample with CPPPS presents moderate levels in the severity of the symptoms. To be precise, it shows moderate levels in the subscales of pain and moderate to severe in urinary symptoms and quality of life. The conditions of physical and psychological health indicate a worse functioning than in the general population; the levels of depression and anxiety are slight, those of pain catastrophizing are between slight and moderate and those of social support are high. The consumption of analgesics (B = 3.78; *p* = 0.006), the presence of depressive symptoms (B = 0.40; *p* = 0.01) and pain catastrophizing (B = 0.18; *p* = 0.001) are significantly associated with a greater severity of the symptoms of CPPPS; while high levels of depressive symptoms are significantly associated with a deteriorated quality of physical (B = −0.85; *p* < 0.001) and psychological (B = −0.63; *p* < 0.001) life.
Stasi et al. (2019)	*n* = 150	Male = 35 (23.3%) Female = 115 (76.4%) Mean age = 41	IBS Symptom Severity Scale (IBS-SSS) - Structured Clinical Interview for DSM-IV (SCID-I) Clinical Global Impressions scale (CGI) - Hamilton Rating Scale for Anxiety (HAM-A) - Montgomery-Asberg Depression Rating Scale (MADRS) - Symptom Check list – 90 (Revised) [SCL-90-R] - Quality of Life Enjoyment and Satisfaction Questionnaire (Q-LES-Q) Arizona Sexual Experience Scale (ASEX)	The sample with IBS presents moderately severe gastrointestinal symptoms. Sixty-nine percent have no diagnosis from the Axis-I, while 30.7% have one or several. The main diagnoses are panic disorder (17.4%), major depression (14.7%), anorexia nervosa (3.3%) and generalized anxiety disorder (2.7%). Furthermore, the sample presents slight to moderate levels of anxiety, depression, compulsive obsession and somatization. The quality of life is slightly satisfactory and no deterioration was found in sexual behavior.
Bouchoucha et al. (2020)	*n* = 608 - IBS (235) - Control (373)	Male = 189 (31%) Female = 419 (69%) Mean age=44.5	Rome III Criteria for IBS Self-report of recurrent abdominal pain or discomfort Minnesota Multiphasic Personality Inventory-2 (MMPI-2)	The group with IBS presents significantly higher symptom exaggeration (*p* < 0.001), hypochondria (*p* < 0.001), depression (*p* < 0.001), hysteria (*p* < 0.001), psychopathic deviation (*p* = 0.005), masculinity-femininity (*p* < 0.001), paranoia (*p* < 0.001), psychasthenia (*p* < 0.001), schizophrenia (*p* < 0.001), hypomania (*p* = 0.005) and social introversion (*p* = 0.042) than the control group. - The IBS patients were classified in the subtypes IBS-Constipation (*n* = 77), IBS-Diarrhea (*n* = 68), IBS-Mixed (*n* = 54) and IBS-Not specified (*n* = 36). The subtypes IBS-Constipation, IBS-Diarrhea and IBS-Mixed present significantly higher levels of hypochondria (*p* < 0.001/ *p* < 0.001/ *p* < 0.001) and depression (*p* < 0.013/ *p* < 0.005/ *p* < 0.001) than the control group. The subtype IBS-Mixed presents significantly higher levels of psychasthenia (*p* < 0.001) and hypomania (*p* = 0.004) than the control group.
Klotz et al. (2020)	*n* = 187	Male = 81 (43.3%) Female = 106 (56.7%) Mean age = 49.06	Short-form McGill Pain Questionnaire (SF-MPQ) Pain Catastrophizing Scale (PCS) NIH-Chronic Prostatitis Symptom Index (NIH-CPSI) Modules of the Patient Health Questionnaire (PHQ-D): Stress module (PHQ-stress) Patient Health Questionnaire Depression Scale (PHQ-9) Generalized Anxiety Disorder Scale (GAD-7) Patient Health Questionnaire (PHQ-15) for the evaluation of somatic symptom severity 12-Item Short Form Health Survey (SF-12)	The sample with CPPPS presents slight to moderate levels of anxiety, depression and stress. Similarly, it presents moderate levels of somatic symptoms and slight to moderate levels of pain catastrophizing. The conditions of physical and psychological health indicate a worse functioning with respect to the general population, as well as slight to moderate levels of pain on sensorial and affective levels.
Porcelli et al. (2020)	*n* = 203 - IBS– Moderate Symptoms (110) - IBS– Severe Symptoms (93)	Male = 58 (28.6%) Female = 145 (71.4%) Mean age = 33.7	Structured Interview for Diagnostic Criteria for Psychosomatic Research - Revised (DCPR-R) Diagnosis of Somatic Symptom Disorder (SSD): Patient Health Questionnaire (PHQ-12) for the evaluation of somatic symptom severity - Modified version of the PHQ-15 Whiteley Index-7 (WI-7) IBS Symptom Severity Scale (IBS-SSS) Hospital Anxiety and Depression Scale (HADS) 12-Item Short Form Health Survey (SF-12)	In the sample with IBS, 27.1% obtained a diagnosis of Somatic Symptoms Disorder (SSD); while 89.7% obtained at least one diagnosis of Psychosomatic Disorders (DCPR-R) and 68.5% more than one. 20.2% fulfilled the criteria for the diagnosis of both disorders. - The group with IBS-Severe Symptoms is significantly older (*p* = 0.003), presents higher upper levels of depression (p =0.01) and anxiety (p =0.01), and presents a greater deterioration in the psychosocial functioning (physical health: *p* = 0.02; psychological health: *p* = 0.02) in comparison to the group with IBS-Moderate Symptoms. The diagnosis of DCPR-R was significantly higher in the group with IBS-Severe Symptoms (96.8%) (*p* = 0.01) in comparison to the group with IBS-Moderate symptoms (61.8%), with significant differences in alexithymia (*p* < 0.001) and persistent somatization (*p* = 0.001). The diagnosis of DCPR-R, to be precise, of alexithymia and persistent somatization, independently and significantly predict the severity of the IBS by explaining part of the covariance of the IBS (18.5%), with a size effect of great magnitude (d = 1.18).
Torun et al. (2020)	*n* = 104 - IBS (54) - Control (50)	Male=35 (33.6%) Female = 69 (66.4%) Mean age=38.78	Symptom Check list – 90 (Revised) [SCL-90-R] State-Trait Anxiety Inventory (STAI)	The group with IBS presents significantly high levels of somatization (*p* < 0.001), compulsive obsession (*p* < 0.001), hostility (*p* < 0.026) and anxiety trait (*p* < 0.001) in comparison to the control group. - The IBS patients with antecedents of physical or psychological trauma present significantly high levels of somatization (*p* < 0.001), compulsive obsession (*p* < 0.001), interpersonal sensitivity (*p* < 0.001), depression (*p* < 0.001), anxiety (*p* < 0.001), hostility (*p* < 0.001), phobias (*p* < 0.001), paranoid ideation (*p* < 0.001) and psychoticism (*p* < 0.001) with respect to those without such antecedents. - The IBS patients with psychological disorders in first degree family members present significantly high levels of compulsive obsession (*p* = 0.002), interpersonal sensitivity (*p* = 0.015), depression (*p* < 0.001), anxiety (*p* < 0.001), hostility (*p* = 0.015), phobias (*p* = 0.030), paranoid ideation (*p* < 0.001) and psychoticism (*p* = 0.017) with respect to those without. - The IBS patients with major stress at the start of the pain present significantly high levels of somatization (*p* < 0.001), compulsive obsession (*p* < 0.001), interpersonal sensitivity (*p* = 0.032), depression (*p* = 0.034), anxiety (*p* = 0.005), hostility (*p* = 0.002), phobias (*p* = 0.046), paranoid ideation (*p* < 0.022) and a state of anxiety (*p* < 0.001) with respect to those who do not.
Gogheri et al. (2021)	*n* = 472 - IBS (236) - Control (236)	Male = 165 (35%) Female = 307 (65%) Mean age = 38.9	Rome III Criteria for IBS Beck Depression Inventory-short Form (BDI-13) Type D Personality Scale (DS-14) Interpersonal Cognitive Distortions Scale Family functioning Assessment Device (FAD)	The group with IBS present slight to moderate levels of depression, as well as significant differences in social inhibition (*p* < 0.001), negative affectivity (*p* = 0.014), interpersonal cognitive distortions (*p* < 0.001) and in family functioning (*p* < 0.001) with respect to the control group. The coefficient of the indirect path between the type D personality and the depression explained through the cognitive distortions (indirect coefficient = 0.11) and the family functioning (indirect coefficient = 0.08) is significant.

**Table 3:** Neuropsychological disorders.

**References**	**Sample**	**Gender and age**	**Instruments**	**Results**
Aizawa et al. (2012)	*n* = 60 - IBS (30) - Control (30)	Male = 30 (50%) Female = 30 (50%) Mean age = 21.6	Rome III Criteria for IBS Structured Clinical Interview for DSM-IV (SCID-I) Wechsler Adult Intelligence Scale-Revised (WAIS) Wisconsin Card Sorting Test (WCST) – Computerized version Functional magnetic resonance imaging (fMRI)	The group with IBS presents significantly more errors of perseverance (*p* = 0.049) and difficulties to maintain the series (*p* = 0.012) in the WCST than the control group. The group with IBS presents significantly lower activity in the right dorsolateral prefrontal cortex (*p* < 0.001) and in the right hippocampus (*p* < 0.001), as well as significantly greater activity in the left posterior insular (*p* < 0.001) when advised of the error in the change of the series, thus showing evidence of deficits in cognitive flexibility. The causal model during the change of series indicates that the subjects with IBS present a significantly inferior connectivity (*p* = 0.012) between the right dorsolateral prefrontal cortex and the supplementary pre-motor area than the control group.
Berrill et al. (2013)	*n* = 231 - IBS (40) - IBD (150) Control (41)	Male = 84 (36.4%) Female = 147 (63.6%) Mean age = 42.5	Rome III Criteria for IBS Hospital Anxiety and Depression Scale (HADS) IBS Symptom Severity Scale (IBS-SSS) Cardiff Cognitive Battery	The groups with IBS and IBD present significantly higher scores in anxiety (*p* < 0.001) and depression (*p* < 0.001) with respect to the control group. Despite having a lower performance in the fluid and crystallized intelligence tests, the group with IBS did not present significant differences with the control group. Nor were significant differences found in episodic memory, working memory, interference, psychomotor speed or attention.
Kennedy et al. (2014)	*n* = 97 - IBS (39) - Crohn's disease (18) - Control (40)	Male = 28 (28.9%) Female = 69 (71.1%) Mean age=29.2	Rome III Criteria for IBS Hospital Anxiety and Depression Scale (HADS) Patient Health Questionnaire Depression Scale (PHQ-9) Cambridge Neuropsychological Test Automated Battery (CANTAB): Paired Associates Learning test (PAL) Intra-Extra Dimensional Set Shift test (IED) Spatial Working Memory test (SWM) Stroop test - Computerized version	The group with IBS was classified into the subtypes IBS-Constipation (*n* = 4), IBS-Diarrhea (*n* = 7) and IBS-Mixed (*n* = 28). The patients with IBS obtained a significantly higher score in depression (*p* < 0.001) and anxiety (*p* < 0.001) with respect to the control group. The group with IBS obtained a significantly deteriorated performance in visuospatial episodic memory (*p* = 0.03) with respect to the control group, but not with respect to the group with Crohn's disease (*p* =0.97). The group with IBS obtained results close to being significant in inhibiting interference and selective attention (*p* = 0.06). In cognitive flexibility, formation, change and maintenance of attention sets and working memory, no significant differences were found between the groups. Furthermore, in the IBS group, the levels of depression and anxiety interfered significantly with the performance in visuospatial memory (*p* = 0.03), but not in the tests for selective attention and response inhibition (*p* = 0.129).
Phillips et al. (2014)	n = 41 - IBS (21) - Control (20)	Male = 9 (22%) Female = 32 (78%) Mean age = 32	IBS Symptom Severity Scale (IBS-SSS) Visual Analog Scales (VAS): The vigor scales: VAS-V1 (tired–energetic) and VAS-V2 (active–drowsy). The stress scales: VASS1 (tense–peaceful) and VAS-S2 (relaxed–worried) Stroop test – Emotional version	The group with IBS presents moderately severe gastrointestinal symptoms. At baseline, they are significantly more tired (*p* = 0.003), sleepy (*p* = 0.001) and tense (*p* = 0.05) than the control group. The group with IBS, in general, presents significantly fewer correct responses in the emotional Stroop test (*p* = 0.005); to be precise, in gastrointestinal (*p* = 0.024) and negative emotional (*p* = 0.016) words in comparison to the control group. The logistic regression model indicates that the subjective and cognitive factors are significantly related to the IBS (χ2 = 23.67; *p* < 0.001), accurately categorizing 85% of the participants. Similarly, the severity of the IBS symptoms correlates negatively with somnolence (r = −0.479) and tiredness (r = −0.440).
Tkalcic et al. (2014)	*n* = 55 - IBS (27) - Control (28)	Male = 9 (16.4%) Female = 46 (83.6%) Mean age = 42.1	Rome III Criteria for IBS State-Trait Anxiety Inventory (STAI) Big Five Inventory (BFI) Visceral Sensitivity Index (VSI) Stroop test – Emotional version Global/Local Task	The group with IBS present significantly higher levels of neuroticism (*p* < 0.05), anxiety trait (*p* < 0.05) and specific visceral anxiety (p < 0.05) than the control group. This group presents two different attention biases: - The global precedence index (precedence of global as opposed to local attention) correlated negatively with neuroticism (r = −0.41; *p* < 0.05), but not with trait or visceral anxiety. - Stroop's facilitation index (response latency) for situational threatening words correlated positively with trait anxiety (r = 0.43; *p* < 0.05) and visceral anxiety (r = 0.47; *p* < 0.05); while the facilitation index for emotional words correlated positively with neuroticism (r = 0.40; *p* < 0.05).
Farup and Hestad (2015)	*n* = 66 - Idiopathic depression (47) - With IBS (27) - Without IBS (20) - Non-organic neurological symptoms (19) - With IBS (6) - Without IBS (13)	Male = 29 (44%) Female = 37 (56%) Mean age = 46	IBS Symptom Severity Scale (IBS-SSS) Beck Depression Inventory-Second Edition (BDI-II) Montgomery-Åsberg Depression Scale (MADRS) Mini-Mental State Examination (MMSE) Trail Making Test (TMT) Grooved Pegboard Test (GPT) Hopkins Verbal Learning Test (HVLT) Brief Visual Memory Test (BVMT) Wechsler Adult Intelligence Scale (WAIS-III) Stroop test Controlled Oral Word Association Test (COWAT)	The patients of the depression group present significantly higher scores for depression (*p* < 0.001/ *p* < 0.001), severity of gastrointestinal symptoms (*p* = 0.003), TMT (A: *p* = 0.01/ B: *p* = 0.02), GPT (*p* = 0.02), Stroop (p =0.003) and COWAT (*p* = 0.008) than the neurological group. In the neurological group, the patients with IBS do not present any significant differences in any of the parameters with respect to the patients without IBS. In the depression group, the patients with IBS present significantly higher scores for depression (*p* = 0.007/ *p* = 0.04) and severity of gastrointestinal symptoms (*p* = 0.02) with respect to the patients without IBS, but there are no differences in the cognitive parameters. The IBS is an independent predictor of depression, but was not associated with any of the cognitive parameters; nevertheless, depression was associated with a deteriorated cognitive performance in attention, cognitive processing (*p* < 0.007), verbal fluency (*p* = 0.005), psychomotor speed (p =0.014/ p =0.017) and set-shifting (*p* = 0.024).
Hubbard et al. (2015)	*n* = 29 - IBS (15) - Control (14)	Female = 29 (100%) Mean age = 31	Rome III Criteria for IBS Hospital Anxiety and Depression Scale (HADS) Visceral Sensitivity Index (VSI) Perceived Stress Questionnaire (PSQ) Temperament and Character Inventory – Revised (TCI-R) Patient Health Questionnaire (PHQ-15) for the evaluation of somatic symptom severity State-Trait Anxiety Inventory (STAI) Attention Network Test (ANT) Functional magnetic resonance imaging (fMRI)	The group with IBS was classified in the subtypes IBS-Constipation (*n* = 7), IBS-Diarrhea (*n* = 4), IBS-Mixed (*n* = 1) and IBS-Not specified (*n* = 3). It presents significantly higher levels of somatic symptoms (*p* = 0.035), visceral anxiety (*p* < 0.001), fear of uncertainty (*p* = 0.048) and perceived stress (*p* < 0.001) than the control group. As for the attention levels, the group with IBS presents a greater effectiveness in alerting (g = −0.34) and in orienting (g = −0.91) attention networks than the control group; while no significant differences were found between both groups in executive control attention networks. In both groups, fear of uncertainty correlated negatively with effective scores in the orienting attention network (r = −0.30) and the high scores of visceral anxiety correlated negatively with effective scores in the alerting attention networks. Within the IBS group, effective scores in alerting attention networks correlated negatively with a greater abdominal pain in the previous week (r = −0.53) and the effective scores in alerting attention networks and executive control attention networks correlated negatively with a greater severity of usual symptoms (r = −0.67/r = −0.51). No correlations were found between the scores of the attention networks and depression, state-trait anxiety or perceived stress.
Chen et al. (2016)	*n* = 161.490 - IBS (32.298) - Control (129.192)	Male = 76.520 (47.4%) Female = 84.970 (52.6%) Mean age = 51.5	Clinical diagnoses from the Taiwan's National Health Insurance Research Database (NHIRD)	The group with IBS presents a significant number of psychological comorbidities (*p* < 0.001) in comparison to the control group. Of these, it is worth highlighting depression (*p* < 0.001). This group presents a greater accumulated incidence of dementia (*p* < 0.001) and a greater risk of dementia (*p* < 0.001) in comparison to the control group. Similarly, the incidence of dementia increases with age and tends to coincide with the incidence of comorbidities, these being significantly higher in the age groups of 50-64 (*p* < 0.001) and >65 (*p* < 0.001), with respect to the group < 49. The patients with IBS present a significantly higher risk of dementia in comparison to the control group in the age groups 50-64 (*p* < 0.01) and >65 (*p* < 0.001), in the female (*p* < 0.01) and masculine (*p* < 0.001) genders, with comorbidities (*p* < 0.001) and without them (*p* < 0.001).
Henrich and Martin (2018)	*n* = 80 - IBS (41) - Control (39)	Male = 13 (16.3%) Female = 67 (83.7%) Mean age = 34.7	Gastrointestinal Symptom Rating Scale for IBS (GSRS-IBS) Gastrointestinal Cognitions Questionnaire (GI-Cognitions) Depression Anxiety Stress Scale (DASS-21) Visceral Sensitivity Index (VSI) Attention Network Test (ANT)	The group with IBS presents significantly higher levels of severity in the gastrointestinal symptoms (*p* < 0.001), catastrophizing cognitions of the IBS (*p* < 0.001), depression (*p* < 0.001), stress (*p* < 0.001), anxiety (*p* < 0.001) and visceral anxiety (*p* < 0.001) with respect to the control group. As for the attention levels, the group with IBS presents a significantly reduced functioning in executive control attention networks (p =0.007) and not significant levels in the attention networks of orienting (*p* = 0.305) and alerting (*p* = 0.232), with respect to the control group. Similarly, the IBS subjects with high scores in reduced attention control present significantly higher levels of catastrophizing cognitions (*p* = 0.032) than those IBS subjects with low scores in reduced attention control.
Wong et al. (2019a)	*n* = 80 - IBS (40): - Constipation Subtype (20) Diarrhea Subtype (20) - Control (40)	Male = 32 (40%) Female = 48 (60%) Mean age = 51.8	Rome III Criteria for IBS Structured Clinical Interview for DSM-IV (SCID-I) Beck Anxiety Inventory (BAI) Beck Depression Inventory-Second Edition (BDI-II) Continuous performance test (CPT) Wisconsin Card Sorting Test (WCST) Stroop test – Emotional version Patient Health Questionnaire (PHQ-15) for the evaluation of somatic symptom severity	The group with IBS presents significantly higher levels of anxiety (*p* < 0.001), depression (*p* < 0.001) and severity of somatic symptoms (*p* < 0.001) than the control group. Around 45% of the subjects of the IBS group fulfill the criteria for the generalized anxiety disorder. In the CPT, the group with IBS showed a significantly higher standard deviation in the reaction time (*p* = 0.003) to that of the control group, and in the WCST, it showed greater errors of perseverance (*p* = 0.003) and greater faults in maintaining the series (*p* = 0.002) than the control group. In the emotional Stroop, despite the results of the IBS group being worse, there are no significant differences with the control group. According to the logistic regression model, the results of the BAI, BDI-II, PHQ-15 and the standard deviation in the reaction time in the CPT (AOR = 1.08; *p* = 0.025) are significantly related to the IBS. Nevertheless, there are no correlations between the IBS patients with symptoms of depression and anxiety and the cognitive parameters.
Rustamov et al. (2020)	*n* = 18 - IBS (9) - Control (9)	Female = 18 (100%) Mean age = 28.7	36-Item Short Form Health Survey (SF-36) Pain Catastrophizing Scale (PCS) Pain vigilance and awareness questionnaire (PVAQ) Beck Depression Inventory-Second Edition (BDI-II) State-Trait Anxiety Inventory (STAI) St-Luc Gastrointestinal Index (GI) Stroop test – Modified version Continuous electroencephalogram (EEG) Visual Analog Scale (VAS)	The group with IBS presents significantly lower levels of physical (*p* < 0.001) and psychological (*p* < 0.008) health than those of the control group, as well as significantly higher levels of pain vigilance (*p* < 0.01) and pain catastrophizing (*p* < 0.02) than those of the control group. In the Stroop test, the IBS group presented higher reaction times (*p* = 0.054) than those of the control group during the inhibition trials, although the differences were not significant. In the denomination trials, the reaction time of the IBS group (p < 0.10) and the control group did not differ significantly. The results of the counter-stimulation and the distraction measured through the encephalogram indicate that the group with IBS presents disorders in pain inhibition, which reflect the interaction between diverse deficits in the processes in the brain related to pain and selective attention.
Sharma et al. (2021)	*n* = 90 - IBS (31) - Ulcerative colitis (29) - Control (30)	Male = 83 (92.2%) Female=7 (7.8%) Mean age = 39.9	Rome III Criteria for IBS Mini-Mental State Examination (MMSE) Montreal Cognitive Assessment (MoCA) test P300 Evoked Potential Protocol	In the MMSE, the group with IBS presents a slightly deteriorated performance (*p* = 0.51), suggestive of a slight cognitive deterioration, although there are no significant differences with the control group. In the MoCA, the group with IBS presents a slightly deteriorated performance (*p* = 0.89), suggestive of a slight cognitive deterioration, although there are no significant differences with the control group. As for the p300 protocol, there are no significant differences in the mean peak amplitude of the p300 wave between the IBS group, the IBD group and the control group (*p* = 0.06), or in the mean peak latency of the p300 wave between the IBS group and the control group (*p* = 0.52).

The CPVP diagnosis was made by a health professional in a total of 31 articles (94%), and in the other 2 articles, although the research was carried out in the health context, it was not specified. As for the CPVP subtypes present in the review; the dominant one is the irritable bowel syndrome that appears in 29 of the articles (88%), followed by the chronic primary pelvic pain syndrome (12%). Of those studies whose main pathology is the irritable bowel syndrome, 7 of them (23%) classified the sample in accordance with the subtypes IBS-Constipation (32.1%), IBS-Diarrhea (31.8%), IBS-Mixed (29%) and IBS-Not specified (7.1%).

As for the presence of a control group, most of the studies use a healthy control group with no diagnosis (67%), while the rest use only the one group (33%) made up of subjects with the pathologies being investigated. Of the studies with a control group, four (18%) include an additional group with another gastrointestinal pathology to compare with the irritable bowel syndrome, to be precise: inflammatory bowel disease (9%), Crohn's disease (4.5%) and ulcerative colitis (4.5%).

### Number, gender and age of the participants

The total number of participants evaluated in the reviewed studies is 255,068, these being 18 participants from the smallest sample (Rustamov et al., 2020) and 161,490 participants from the largest (Chen et al., 2016). The proportion of female (51%) and male (49%) participants is balanced, and the mean age is 40.5 years (SD = 7.9), with a range between 18 and 85 years of age.

It should be stated that those participants diagnosed as suffering from CPVP totalled 69,395 (27.21%) and, as with the total number of participants, the proportion of females (52%) and males (48%) is balanced and the mean age is 40.5 years (SD = 7.9).

### Instruments for evaluation and variables of the CPVP, psychopathological and neuropsychological

As can be seen in Tables 2, 3, the instruments used in the reviewed studies present a great variability, with a total of 67 different instruments to evaluate the subtypes of CPVP and the psychopathological and neuropsychological disorders. In general, the Rome III Criteria for IBS (8%), the IBS Symptom Severity Scale (IBS-SSS) (6%), the Beck Depression Inventory (BDI) (4%), the Patient Health Questionnaire Depression Scale (PHQ-9) (4%) and the Stroop test (4%) were the most commonly used instruments. These instruments were used to evaluate a total of 66 different variables, of which the most commonly evaluated were: depression (10%), anxiety (9%), the symptoms of irritable bowel syndrome (6%), the severity of the symptoms of IBS (5%) and the inhibitive capacity (4%).

As for CPVP and its subtypes, a total of 13 of the instruments (19.4%) were aimed at its evaluation; the most commonly used being the Rome III Criteria for IBS (25%), the IBS Symptom Severity Scale (IBS-SSS) (21%), the Pain Catastrophizing Scale (PCS) (10%), the NIH-Chronic Prostatitis Symptom Index (NIH-CPSI) (8%) and the Visual Analog Scale (VAS) (8%). These instruments evaluated a total of 16 different variables (24.2%), the most commonly evaluated being: the symptoms of irritable bowel syndrome (22%), the severity of the symptoms of irritable bowel syndrome (20%), pain catastrophizing (9%), somatic symptoms (9%) and the specific distress of the gastrointestinal symptoms (5%).

As for the psychopathological disorders, 37 of the instruments (55.2%) were aimed at their evaluation, the most commonly used being: the Beck Depression Inventory (BDI) (7%), the Patient Health Questionnaire Depression Scale (PHQ-9) (7%), the Generalized Anxiety Disorder Scale (GAD-7) (6%), the Structured Clinical Interview for DSM-IV (SCID) (6%) and the Hospital Anxiety and Depression Scale (HADS) (5%). These instruments evaluated a total of 25 different variables (37.9%), the most commonly evaluated being: depression (24%), anxiety (22%), psychopathological symptomatology (9%), the quality of life related to health (6%) and the DSM-IV disorders (6%).

As for the neuropsychological disorders, 17 of the instruments (25.3%) were aimed at their evaluation, the most commonly used being: the Stroop test (23%), the Wisconsin Card Sorting Test (WCST) (8%), the Mini-Mental State Examination (MMSE (8%), the Attention Network Test (ANT) (8%) and the Wechsler Adult Intelligence (WAIS) (8%). These instruments evaluated a total of 25 different variables (37.9%), the most commonly evaluated being: attention (33%), inhibition (13%), the processing of information (9%), work memory (6%) and cognitive flexibility (6%).

### Results of the evaluation of the CPVP, of the psychopathological and neuropsychological disorders

#### Chronic primary visceral pain

The severity of the CPVP and its subtypes were evaluated in a total of 18 articles (55%). The majority of the participants with this pathology presented moderate or severe thoracic, abdominal and/or pelvic symptoms. In particular, in the study of Lackner et al. (2013), the severity of the said symptoms was high. Concerning these symptoms, the pain catastrophizing and light to moderate levels of the somatic symptoms related to this disease should be highlighted (each one present in 28% of these articles), as well as the specific distress linked to the gastrointestinal symptoms (present in 17%) and the psychological anxiety also linked to the gastrointestinal symptoms (present in 6%). Other studies evaluated the general state of health (33% of these articles), showing evidence on the whole of a deteriorated physical and psychological functioning. Only Lackner et al. (2013) and Thakur et al. (2016) evaluates the quality of life related to the disease, obtaining a result indicating deterioration as a consequence of the gastrointestinal symptoms.

#### Psychopathological disorders

The psychopathological disorders of CPVP and its subtypes were evaluated in a total of 28 articles (85%). The psychological disorders with the greatest prevalence in the participants with this pathology, diagnosed in 9 of these articles (33%), were: those of anxiety, to be precise, generalized anxiety disorder (present in 25% of these articles); mood disorders, to be precise, major depressive disorder (present in 18%); and somatoform disorders (present in 14% of these articles); followed by bipolar disorder (present in 7%) and panic disorder, post-traumatic stress disorder, obsessive-compulsive disorder, sleep disorders, substance use disorders, anorexia nervosa, borderline personality disorder, dependent personality disorder, histrionic personality disorder and narcissistic personality disorder (each one present in 4%). It is worth highlighting the study of Liu et al. (2015), which focuses solely on evaluating the overall incidence rate and the risk of suffering bipolar disorder in a population with irritable bowel syndrome; the results were significantly higher than in the general, healthy population.

The psychopathological symptoms with the greatest prevalence in CPVP, evaluated in 22 of these articles (79%), were: moderate levels of anxiety (present in 46% of these articles), depression (present in 39%) and stress (present in 18%), followed by somatization, compulsive obsession, hostility, psychoticism (each one present in 7%) and interpersonal sensitivity, phobias, paranoid ideation, insomnia, anxiety due to illness, adverse childhood experiences, mentalizing deficits, hypochondria, neuroticism, hysteria, social inhibition, interpersonal cognitive distortions and fear of uncertainty (each one present in 4%). Only Lackner et al. (2013), Dybowski et al. (2018) and Piontek et al. (2019) evaluated the quality of life of the participants with this pathology, obtaining as a result a significant deterioration in the said quality of life.

#### Neuropsychological disorders

The neuropsychological disorders of CPVP and its subtypes were evaluated in a total of 12 articles (36%). The executive functions, evaluated in 11 of these articles (92%), show uneven results. Aizawa et al. (2012) and Wong et al. (2019a), through the Wisconsin Card Sorting Test (WCST), show evidence of deficiencies in cognitive flexibility and in problem solving in the participants with this pathology. They have significantly more errors of permanence and difficulties to maintain the series than the control group; meanwhile, Kennedy et al. (2014), through the Intra-Extra Dimensional Set Shift test (IED), found no significant differences in cognitive flexibility in a comparison of both groups. Kennedy et al. (2014), Phillips et al. (2014) and Wong et al. (2019a), through the Stroop test (computerized and emotional versions), show evidence of deficiencies in response inhibition and cognitive processing of emotional content in the participants with CPVP; meanwhile, Rustamov et al. (2020), through the Stroop test–Modified version, shows for this group insignificant reaction times in inhibition, but still higher than those of the control group. Similarly, Berrill et al. (2013) and Kennedy et al. (2014) found no significant differences in processing speed and work memory, respectively, in comparison to the control group.

Attention, evaluated in 8 of these articles (67%), shows congruent results. Aizawa et al. (2012) and Wong et al. (2019a), through the Wisconsin Card Sorting Test (WCST), show evidence in the participants con CPVP of deficits in this cognitive domain. On evaluating the different attention networks in an individualized manner, Hubbard et al. (2015) and Henrich and Martin (2018), using the Attention Network Test (ANT), showed evidence that the participants with this pathology had a slightly better efficiency in orientation attention networks and warning attention networks (sustained attention) in comparison to the control group; while in the executive control attention networks (selective attention) they showed a reduced functioning in comparison to the control group. These results are coherent with those of Kennedy et al. (2014), Phillips et al. (2014) and Wong et al. (2019a), which showed deficits, though mostly insignificant, in the selective attention of the participants with CPVP.

In intelligence, evaluated in 2 of these articles (17%) using the Wechsler Adult Intelligence Scale (WAIS), despite the fact that there were no significant differences with respect to the control group; there was evidence of a lower performance level in the fluid and crystallized intelligence tests of the participants with CPVP. In memory, evaluated in 2 of these articles (17%), the results were uneven. Kennedy et al. (2014), using the Paired Associates Learning test (PAL), showed evidence in the participants with this pathology of a significantly deteriorated performance in episodic visuospatial memory with respect to the control group; however, Berrill et al. (2013), using the Cardiff Cognitive Battery, showed very similar results in episodic visuospatial memory in both groups.

Chen et al. (2016) evaluate the risk of dementia in CPVP, showing evidence of a greater accumulated incidence of dementia and a greater risk of dementia with the irritable bowel syndrome in comparison to the control group. Similarly, Sharma et al. (2021), using the Mini-Mental State Examination (MMSE) and the Montreal Cognitive Assessment (MoCA) test, showed evidence of a slight cognitive deterioration in participants with these pathologies in comparison to the control group.

## Discussion

The present study has carried out the first systematic review of the psychopathological and neuropsychological disorders associated with CPVP, in accordance with the new conceptualization of chronic pain from the WHO and the International Association for the Study of Pain (IASP) in the last revision of the ICD-11 (World Health Organization, 2018). Following an extensive search in the different databases and verification of their suitability for the objectives of the present study, a total of 33 articles were included in this current review.

In general, the studies that evaluate the severity of this pathology conclude that the severity of the thoracic, abdominal and/or pelvic symptoms is frequently considered to be between moderate and severe. Among those who suffer it, they usually have symptoms of pain catastrophizing, somatic symptoms, anxiety in the face of gastrointestinal symptoms, psychological distress, a deteriorated physical and psychological health and a low quality of life as a consequence of the illness (Lackner et al., 2013; Thakur et al., 2016).

Those articles that evaluate the psychopathological disorders of CPVP present similar results. The majority of the participants evaluated present at least one psychological disorder and a considerable percentage even have two (Lackner et al., 2013; Brünahl et al., 2017; Kawoos et al., 2017), the most common being anxiety disorders, depressive disorders and somatoform disorders. Correspondingly, anxiety, depression, somatization, compulsive obsession, and hostility are to be found among the commonest psychopathological symptoms, with moderate intensities. Examining the existing correlations throughout the articles, Lackner et al. (2013), van Tilburg et al. (2013), Thakur et al. (2016), Lee et al. (2017), Dybowski et al. (2018) and Piontek et al. (2019) show that physical and psychological comorbidities, high levels of anxiety, high levels of depression, insomnia, severity of the pain, pain catastrophizing, somatization and age are all significant predictors of a greater severity of the symptoms CPVP, of the evolution of the pain and of a deteriorated quality of life. Similarly, Farup and Hestad (2015), Banerjee et al. (2017) and Porcelli et al. (2020) all show that the level of severity of the CPVP is a predictor of the severity of the associated psychopathological disorders. The greater the severity of the CPVP, the greater the severity of the levels of anxiety and depression, and the worse the psychosocial functioning, the quality of sleep and the conditions of physical and psychological health will be.

Conversely, several works of research have suggested that chronic pain is associated with a deterioration in attention, memory, intelligence and executive functioning (Berryman et al., 2014; Kennedy et al., 2014; Corti et al., 2021); in particular, within this latter domain, with the processes controlling response inhibition, working memory, cognitive flexibility, planning, problem solving and decision taking. Nevertheless, the findings concerning the neuropsychological disorders of the CPVP demonstrated in this current review point toward a great diversity that can even be contradictory.

The findings in executive functioning and memory are the ones that show the greatest ambiguity, particularly in the domains of cognitive flexibility, response inhibition and episodic visuospatial memory. Notwithstanding the above, deficits were found in the domains of problem solving and cognitive processing of emotional content, while the speed of processing and working memory did not seem to suffer any deterioration in the population in question. Although none of the studies reviewed showed correlations between the symptoms of depression and anxiety in CPVP and the results in the cognitive domains; Farup and Hestad (2015) and Wong et al. (2019a) did show evidence that the presence of depression and/or anxiety can act as predictors of a deteriorated cognitive performance in attention, cognitive processing, verbal fluency, psychomotor speed and cognitive flexibility.

The findings in attention are the ones that show the greatest agreement. Although the studies show evidence of deficits in this domain, on making an individualized evaluation of the different attention networks, they do show a slightly better effectiveness than that of the general population in the attention networks of both orienting and alerting (sustained attention); while in the attention networks of executive control (selective attention), there is evidence of a reduced functioning. On examining the existing correlations, Tkalcic et al. (2014) showed a positive attention bias, with a greater latency in the response toward words related to threatening situations and negative emotional content. A positive correlation can be appreciated with the anxiety trait, specific visceral anxiety and neuroticism. Similarly, Hubbard et al. (2015) and Henrich and Martin (2018) found that the fear of uncertainty and the high severity of the usual symptoms of CPVP negatively correlate with effective scores in the orienting attention network; that the high scores of visceral anxiety, the presence of major abdominal pain in the previous week and the high severity of the usual symptoms of CPVP correlate negatively with effective scores in alerting attention networks; and that the high levels of catastrophizing cognitions correlate negatively with a reduced attention control. Lam et al. (2019) and Wong et al. (2019b) state that one of the principal explanations for the demonstrated biases in the attention network is the presence of chronic thoracic, abdominal and/or pelvic pain, anxiety related to the symptoms, hypervigilance of the pain and visceral hypersensitivity. Along the same lines, another possible explanation for the attention bias is the proposal made by Rustamov et al. (2020), which suggests that persons with chronic pain direct a great part of their attention resources to information related to the pain due to their inability to inhibit it.

As for the findings concerning intelligence, although there were no significant differences with respect to the general population, there was evidence of a lower performance in fluid intelligence and crystallized intelligence. It can be seen that the risk of dementia has only been evaluated in one study (Chen et al., 2016); the accumulated incidence and the risk of suffering dementia being greater in patients with chronic pain. The incidence of dementia increases with age in the population with this pathology and tends to coincide with the incidence of comorbidities, being significantly higher from fifty years of age upwards.

The current review has some limitations. The conceptualization of chronic primary pain and, in particular, of CPVP and its subtypes is very recent, which influences the fact that the majority of studies found in the databases have not been adapted to this classification and that, therefore, the number of studies is greatly reduced. Thus, the only subtypes included in this present review are the irritable bowel syndrome and the chronic primary pelvic pain syndrome.

The control of the comorbidity of the participants included in the review is a limitation. Although most of the exclusion criteria refer to the presence of other gastrointestinal diseases, organic diseases, serious medical conditions, serious psychological disorders before the diagnosis of CPVP, substance abuse and/or the consumption of certain medications, there is great heterogeneity between the different articles. This can cause some articles to consider variables that others do not, affecting the validity of the results.

The instruments used are another limitation. The reviewed studies use a great number of different instruments to evaluate the psychopathological and neuropsychological disorders of the participants, which complicates the comparison of the results of the different studies. Not all the studies evaluate the disorders present in the sample in the same way; since some focus on psychopathological disorders, others on neuropsychological disorders and yet others focus on both. Furthermore, while some studies evaluate the general disorders, others focus on particular domains. All this has an impact in the sense that some results, especially those related to the neuropsychological disorders, can be ambiguous and, to a certain extent, contradictory. Similarly, the relations between the variables shown in the different studies are also very diverse; in some cases, resulting in difficulties to relate the psychopathological and neuropsychological disorders with CPVP.

Likewise, the use of self-reports or clinical interviews can show disparate results (Stuart et al., 2014). Through the use of self-reports, the presence of psychopathological symptoms is evaluated; however, factors such as lack of understanding, social stigma, the influence of mood, poor memory, and social desirability can influence the results; while the clinical interview, despite being more complex and requiring more time to apply, is considered the gold standard for in-depth evaluation of the presence of psychological disorders (Hopwood et al., 2008; Stuart et al., 2014). Therefore, the importance of using the two procedures for adequate psychopathological evaluation (Hopwood et al., 2008).

There are also several limitations in connection to the sample. Eleven of the studies do not have a control group with which to compare the results of the evaluation and two have a CPVP sample size below twenty subjects, which has repercussions on the reliability of the results. Furthermore, although the mean age is similar in the majority of the reviewed studies, there are some cases in which the age range is wider, which may suppose a limitation.

In short, the current review describes and critically analyses the psychological disorders associated with CPVP. Despite the great scarcity of studies concerning this pathology in the scientific literature, the questions related with psychopathological and neuropsychological disorders have been debated and questioned, concluding that the subjective, emotional and cognitive factors are intimately related to CPVP. Nevertheless, some deficits, especially neuropsychological ones, have not been sufficiently evidenced throughout the reviewed studies, or they show ambiguous results.

For future research, we highlight several objectives. First, to widen the sample to other subtypes; second, to use homogeneous tests to identify associated psychopathological and neuropsychological disorders. Third, we consider it necessary to use homogeneous inclusion and exclusion criteria in the selection of the sample to avoid the presence of comorbidity that could affect the validity of the results. The recent classification of chronic pain and its diverse classifications suppose a starting point for the research to deal with this pathology therapeutically, giving us the possibility of developing specific treatments adapted to the existing disorders.

## Data availability statement

The original contributions presented in the study are included in the article/supplementary material, further inquiries can be directed to the corresponding author.

## Author contributions

AA-M, JM-M, MG-B, MB-A, and PC-C: conceptualization, methodology, and writing—review and editing. AA-M, JM-M, MG-B, and MB-A: data curation and supervision. AA-M, JM-M, and PC-C: formal analysis. AA-M, JM-M, MG-B, and PC-C: writing—original draft preparation. All authors contributed to the article and approved the submitted version.

## Funding

Financed jointly by FEDER & Junta de Extremadura funds (Exp. GR21024). The funder was not involved in the study design, collection, analysis, interpretation of data, the writing of this article or the decision to submit it for publication.

## Conflict of interest

The authors declare that the research was conducted in the absence of any commercial or financial relationships that could be construed as a potential conflict of interest.

## Publisher's note

All claims expressed in this article are solely those of the authors and do not necessarily represent those of their affiliated organizations, or those of the publisher, the editors and the reviewers. Any product that may be evaluated in this article, or claim that may be made by its manufacturer, is not guaranteed or endorsed by the publisher.

## Footnotes

^1^The subtypes of CPVP and their different codifications are shown in Figure 1.

## Abbreviations

CPVP: Chronic primary visceral pain
IBS: Irritable bowel syndrome
IBD: Inflammatory bowel disease
CPPPS: Chronic primary pelvic pain syndrome
ICD: International Classification of Diseases
WHO: The World Health Organization.
